# Nabiximols effect on blood pressure and heart rate in post-stroke patients of a randomized controlled study

**DOI:** 10.3389/fcvm.2022.990188

**Published:** 2022-10-28

**Authors:** Gian Marco Rosa, Luca Puce, Laura Mori, Antonio Currà, Francesco Fattapposta, Italo Porto, Nicola Luigi Bragazzi, Carlo Trompetto, Lucio Marinelli

**Affiliations:** ^1^Cardiology Clinic, Department of Internal Medicine and Medical Specialties, University of Genoa, Genova, Italy; ^2^IRCCS Ospedale Policlinico San Martino, Genova, Italy; ^3^Department of Neuroscience, Rehabilitation, Ophthalmology, Genetics, Maternal and Child Health, University of Genoa, Genova, Italy; ^4^Department of Medical-Surgical Sciences and Biotechnologies, A. Fiorini Hospital, Sapienza University of Rome, Latina, Italy; ^5^Department of Human Neurosciences, Sapienza University of Rome, Rome, Italy; ^6^Laboratory for Industrial and Applied Mathematics (LIAM), Department of Mathematics and Statistics, York University, Toronto, ON, Canada

**Keywords:** stroke, THC, CBD, cannabinoid, cerebrovascular disorders, blood pressure, heart rate, Sativex

## Abstract

**Background:**

Cannabinoids may be useful to treat pain, epilepsy and spasticity, although they may bear an increased risk of cardiovascular events. This study aims to evaluate the cardiovascular safety of nabiximols, a cannabis-based drug, in patients with spasticity following stroke, thus presenting an increased cardiovascular risk.

**Methods:**

This is an ancillary study stemming from the SativexStroke trial: a randomized double-blind, placebo-controlled, crossover study aimed at assessing the effect of nabiximols on post-stroke spasticity. Patients were treated with nabiximols oromucosal spray or placebo and assessed before and after two phases of 1-month duration each. Only the phase with the active treatment was considered for each patient who completed the study. The average values of blood pressure (diastolic, systolic, differential) and heart rate from the first 5 days of the phase (lowest nabiximols dosage) were compared to the average values recorded during the last 5 days at the end of the phase (highest nabiximols dosage). Baseline comparisons between gender, stroke type and affected side and correlation between age and blood pressure and heart rate were performed. The study was registered with the EudraCT number 2016-001034-10.

**Results:**

Thirty-four patients completed the study and were included in the analysis. Thirty-one were taking antihypertensive drugs and, among these, 12 were taking beta-blockers. During the study, no arrhythmic events were recorded, blood pressure and heart rate did not show pathological fluctuations, and no cardiovascular or cerebrovascular events occurred. At baseline blood pressure and heart rate were comparable concerning gender, stroke type and affected side. A significant direct correlation emerged between differential blood pressure and age and an inverse correlation between diastolic blood pressure and age. No correlation emerged between systolic blood pressure or heart rate and age. Blood pressure and heart rate did not change during nabiximols treatment compared to the baseline condition.

**Conclusion:**

This ancillary study adds evidence that, in patients who already underwent a cerebrovascular accident, nabiximols does not determine significant blood pressure and heart rate variation or cardiovascular complications. These data support the cardiovascular safety of nabiximols, encouraging more extensive studies involving cannabinoids characterized by slow absorption rates.

## Introduction

The word “cannabis” refers to all products derived from the plant *Cannabis sativa*. Cannabinoids are a group of substances found in the cannabis plant. The main cannabinoids are delta-9-tetrahydrocannabinol (THC) and cannabidiol (CBD). THC is the substance that is primarily responsible for the effects of marijuana on a person’s mental state (1).

It has been demonstrated that cannabinoids, which were previously used only for recreational purposes, can also be utilized in clinical practice to treat various pathologies, especially pain and chemotherapy-induced nausea, epilepsy and spasticity (2, 3).

The effects of all these substances are mediated through the endocannabinoid system via the interaction with the CB receptors, particularly the CB_1_ receptors, which are located in the myocardium, aorta, vascular endothelium, in platelets, and in the central and peripheral nervous system. Conversely, CB_2_ receptors are found in immune cells (4). Since cannabinoids interact with CB_1_ receptors, they exert many cardiovascular effects, as has been shown by various studies conducted both on animal models (5, 6) and on humans (7).

The main cardiovascular effects of cannabinoids can be summarized as: enhanced sympathetic tone, increased catecholamine levels and increased heart rate at lower doses, and bradycardia/hypotension at higher doses, owing to parasympathetic stimulation occurring at these latter doses (8–10). Other cardiovascular effects which may be identified are platelet activation, endothelial dysfunction and oxidative stress (11, 12).

All these effects can be ascribed to THC; by contrast, CBD has been shown to reduce heart rate and blood pressure, improve vasodilation in models of endothelial dysfunction, and reduce inflammation and vascular hyper-permeability in diabetic models (13). Furthermore, in an interesting study, Mathew found that healthy volunteers who experienced orthostatic hypotension after smoking marijuana presented reduced cerebral blood velocity on transcranial Doppler (14). This reduction in cerebral blood flow can increase the risk of ischemic stroke and the likelihood of falls. This effect raises an important concern regarding the effects of marijuana in older subjects. A further effect of smoking marijuana is an increase in the amount of carboxyhemoglobin due to combustion, thereby causing an additional decrease in oxygen supply (15). Furthermore, many case reports have linked marijuana use to procoagulant effects, thus causing thrombus formation and leading to acute myocardial infarction in young adults (16–18). On the other hand, in many reported cases of cannabis-induced acute myocardial infarction, coronary angiography has documented coronary spasm in the absence of major atherosclerotic lesions (19).

In an interesting study, Mittleman et al. showed that marijuana smoking was associated with a 4.8-fold increased risk of myocardial infarction within 1 h of use, though this higher risk appeared to decrease rapidly thereafter (20). Finally, the pharmacokinetics of cannabinoids varies according to the route of administration, the most common route being inhalation via smoking or vaporization (21). As inhalation (*via* smoking or vaporization) is the fastest method of intoxication, the availability of THC is predictable (22). Indeed, plasma THC levels are detectable within a short time (seconds or minutes) after inhalation and reach a maximum after 15–20 min (22). Furthermore, edible forms of marijuana are now gaining popularity among users (23). Edible forms of marijuana often contain very high amounts of THC and, owing to the erratic absorption of oral marijuana, its effects are delayed (23). Oral consumption is slower to take effect, inducing peak levels at 2–3 h, and its effects are less predictable in most cases, leading to higher levels of complications. In a study on health volunteers, inhalation and IV injection elicited similar plasma THC concentrations and clinical effects, and both caused major acute cardiovascular effects, while oral ingestion induced less predictable and delayed peak plasma THC concentrations (23). THC metabolism includes Phase I reactions, mainly consisting of hydroxylation by the hepatic CYP P450, 2C9, 2C19 and 3A4 enzyme system, and Phase II reactions, which involve the oxidation of alcohols and conjugation with glucoronic acid. THC main metabolites are active 11-OH-THC and inactive THC, which are produced by oxidation (23). THC is excreted mostly as hydroxylated and carboxylated metabolites via feces or urine (24, 25). Both slow release from lipid-storage compartments and significant enterohepatic circulation contribute to the long half-life of THC (> 4 days) (25). CBD metabolism is similar to that of THC, but is subject to a significant first-pass effect; unlike THC, a large proportion of the dose is excreted unchanged in the feces (23). When THC and CBD are simultaneously administered, pharmacokinetic interference may arise; CBD partially inhibits the hydroxylation of THC to 11-OH-THC at CYP P450 and slightly slows the metabolism of 11-OH-THC to THC-COOH (23).

Among the currently used synthetic cannabinoids, a combination of THC and CBD has recently been introduced in order to improve pharmacokinetics and pharmacodynamics. Nabiximols, which is a combination of delta-9-tetrahydrocannabinol THC and cannabidiol CBD in a 1:1 ratio, has recently been introduced into the market. This drug is administered via the oromucosal route to treat spasticity, and various studies conducted in animal models have recently analyzed its pharmacokinetics and pharmacodynamics (26, 27). With regard to pharmacokinetics, THC and CBD might (23, 28, 29) reciprocally interact by interfering with their pharmacokinetics. This interaction depends largely on the ratio of the two drugs and their time of administration. Dose-ratio studies in animals have shown that the simultaneous administration of the two drugs produces a response pattern similar to that observed with a lower dose of THC, suggesting that CBD blocks the effects of THC (30). Studies conducted in humans have shown no significant differences from the effects observed in animal studies (31, 32). Recently, a double-blind randomized placebo-controlled crossover pilot study (SativexStroke Trial) was conducted in the Neurorehabilitation Unit of our Polyclinic to assess the efficacy and safety profile of nabiximols in post-stroke spasticity (33). This pilot study demonstrated the lack of efficacy of nabiximols to treat post-stroke spasticity (34). The aim of the current sub-study was to analyze the cardiovascular safety of nabiximols in a cohort of patients affected by post-stroke spasticity.

## Materials and methods

### Study design

This is an ancillary study stemming from the SativexStroke trial: a randomized double-blind, placebo controlled, crossover study aimed at assessing the effect of nabiximols on post-stroke spasticity. It has been performed at the outpatient service for the treatment of spasticity of the Neurorehabilitation Unit, IRCCS Ospedale Policlinico San Martino (Genova, Italy) in accordance with the Declaration of Helsinki and Good Clinical Practice guidelines. The trial has been registered on the EudraCT platform with number 2016-001034-10.

This study lasted 10 weeks and consisted into two phases: phase 1 and 2, both of them lasting 4 weeks. Phase 1 and 2 were separated by a 2-weeks wash-out interval. During each phase, patients had been taking nabiximols and then placebo or vice-versa in a crossover design following 1:1 randomization.

Each patient was examined 4 times during 10 weeks: before (T0) and after (T1) phase 1, as well as before (T2) and after (T3) phase 2.

### Participants

Adult stroke survivors were recruited according to the following inclusion criteria: (1) male or female patients of at least 18 years of age; (2) spasticity secondary to stroke that occurred at least 3 months earlier; (3) CHA2DS2VASc score < 7 assessed by the cardiologist and reflecting acceptable cardiovascular risk; (4) spasticity rated between 1 and 3 on the Modified Ashworth Scale at the level of at least one of the following muscle groups: wrist flexors, elbow flexors, knee extensors, foot plantar flexors; (5) ability (physical ability or supportive person) to comply with the study requirements correctly and to follow the study procedure and restrictions.

Exclusion criteria were: (1) presence of concomitant parkinsonism; (2) significant peripheral nervous system pathology detectable on clinical basis; (3) current smokers; (4) contraindication to treatment with nabiximols; (5) alcohol or drug abuse, including current consumption of cannabis herb or other cannabinoid-based drugs within 30 days prior to study entry; (6) treatment with botulinum toxin injection in the last 4 months; (7) clinically significant impaired renal function or impaired hepatic function at baseline, (8) females of child bearing potential, pregnant or lactating and male patients whose partner is of child bearing potential who are not willing to use effective contraception.

### Procedures

Patients who gave informed consent to participate underwent a preliminary screening visit to ensure that they fulfilled the study selection criteria, followed by a cardiological evaluation (including ECG and echocardiogram) to assess cardiovascular risk.

At the beginning of each phase (T0 and T2), patients were provided with a form to daily record heart rate, blood pressure (systolic/diastolic) and adverse events. Outcome measures were collected and instructions on how to take the oromucosal spray were provided, along with a schedule to gradual increase daily sprays to reach the highest tolerable dose up to a maximum of 12 sprays/day in a 14-days period and then maintain such daily dose until the end of each phase (T1 and T3) (Table 1).

**TABLE 1:** Nabiximols titration scheme.

Day	Number of sprays		
	Morning	Afternoon/Evening	Total per day
1	0	1	1
2	0	1	1
3	0	2	2
4	0	2	2
5	1	2	3
6	1	3	4
7	1	4	5
8	2	4	6
9	2	5	7
10	3	5	8
11	3	6	9
12	4	6	10
13	4	7	11
14	5	7	12

Patients were required to continue all concomitant medications throughout the study.

### Outcomes

The main and secondary outcomes have been reported in the first report (34).

To the aim of this ancillary study, we considered the daily measurement of blood pressure and heart rate as reported by the patients in the provided form. For each patient who completed the study, we considered only the phase with the active treatment. The average values of blood pressure and heart rate from the first 5 days of the phase (while taking the smallest number of nabiximols sprays) were considered baseline T0 values. The average values recorded during the last 5 days at the end of the phase (while taking the highest number of sprays) were considered T1 values. Regarding heart rate, we consider the self-reported average number of beats per minute, regardless of the presence of an arrhythmia.

### Statistical analysis

Cardiovascular parameters (systolic, diastolic and differential blood pressure; heart rate) are reported as mean ± standard deviation. Sprays number is reported as median (range).

Baseline comparisons where performed using Mann-Whitney U test. Baseline correlations between age and cardiovascular parameters and between the number of sprays taken at T1 and T0-T1 difference of blood pressure and heart rate were performed using Spearman rank test. Cardiovascular parameter comparisons between baseline condition and during nabiximols treatment were performed using Wilcoxon signed rank test. This is a pilot study and no preliminary data about cardiovascular parameter variation during nabiximols treatment were available, so power analysis was not performed. For all tests significance level was set at *p* < 0.05.

## Results

Only the 34 subjects who completed nabiximols cycles where included in the study (Table 2).

**TABLE 2:** Baseline patient characteristics.

**Gender**	
Male	24 (71%)
Female	10 (29%)
Age (y)	68 (59–72)
**Stroke type**	
Hemorrhagic	13 (38%)
Ischemic	21 (62%)
**Affected hemisphere**	
Left	13 (38%)
Right	21 (62%)
Time after stroke (y)	4.2 (1.7–5.6)
**ECG findings**	
Slight changes in the	1 (3%)
recovery phase	
Previous acute	1 (3%)
myocardial infarction	
Supraventricular	4 (12%)
ectopic beats	
Ventricular ectopic	2 (6%)
beats	
Right branch block	2 (6%)
Atrial fibrillation	4 (12%)

Values are reported as number (%) or median (Q1–Q3).

Patients taking anti-hypertensive drugs were 31, among these those taking beta-blockers were 12.

### Baseline analysis

Comparing the 24 male and 10 female patients, no difference emerged for baseline diastolic (*U* = 77, *p* = 0.1), systolic (*U* = 113, *p* = 0.8), differential (*U* = 86, *p* = 0.2) and heart rate (*U* = 74, *p* = 0.08).

Comparing the 21 patients who had an ischemic stroke and the 13 who had a hemorrhagic stroke, no difference emerged for baseline diastolic (*U* = 111, *p* = 0.4), systolic (*U* = 131, *p* = 0.8), differential (*U* = 120, *p* = 0.5) and heart rate (*U* = 119, *p* = 0.5).

Comparing the 21 patients with a right and the 13 with a left hemispheric stroke, no difference emerged for baseline diastolic (*U* = 129, *p* = 0.8), systolic (*U* = 102, *p* = 0.2), differential (*U* = 97, *p* = 0.2) and heart rate (*U* = 116, *p* = 0.5) (Table 3).

**TABLE 3:** Cardiovascular parameters at baseline.

	Gender		Stroke type		Affected hemisphere	
	Male (*n* = 24)	Female (*n* = 10)	Ischemic (*n* = 21)	Hemorrhagic (*n* = 13)	Right (*n* = 21)	Left (*n* = 13)
Diastolic BP	78 ± 6	74 ± 9	78 ± 8	75 ± 6	76 ± 6	77 ± 8
Systolic BP	127 ± 14	126 ± 13	126 ± 14	127 ± 13	128 ± 12	123 ± 15
Differential BP	49 ± 13	52 ± 11	48 ± 13	52 ± 12	52 ± 13	46 ± 9
Pulse frequency	69 ± 8	76 ± 9	71 ± 9	72 ± 8	72 ± 9	70 ± 8

Values are reported as mean ± standard deviation.

**FIGURE 1:**
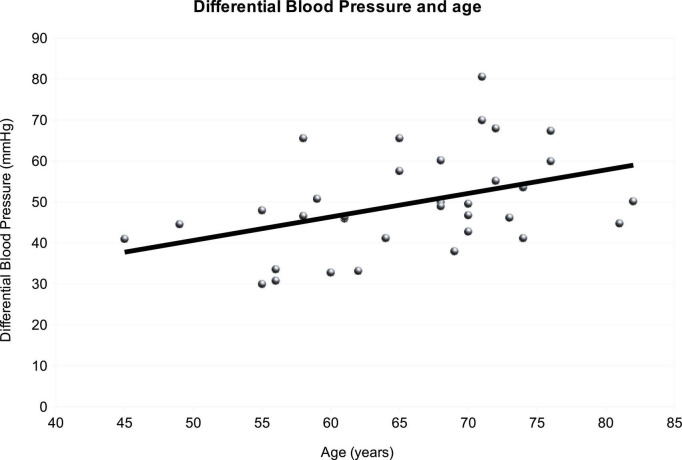
Graphical representation of the relationship between differential arterial pressure and age. With increasing age also differential arterial pressure increases.

Diastolic blood pressure inversely correlated with age (Rho = −0.4, *p* = 0.014), while systolic pressure did not (Rho = 0.1, *p* = 0.4). A significant direct correlation emerged between age and differential blood pressure (Rho = 0.5, *p* = 0.008) (Figure 1). Age and heart rate did not correlate (Rho = −0.07, *p* = 0.7).

### Nabiximols effect

On average, at T1 patients were taking 9 nabiximols sprays (range 1–12), corresponding to 24.3mg of THC and 22.5mg of CBD.

**FIGURE 2:**
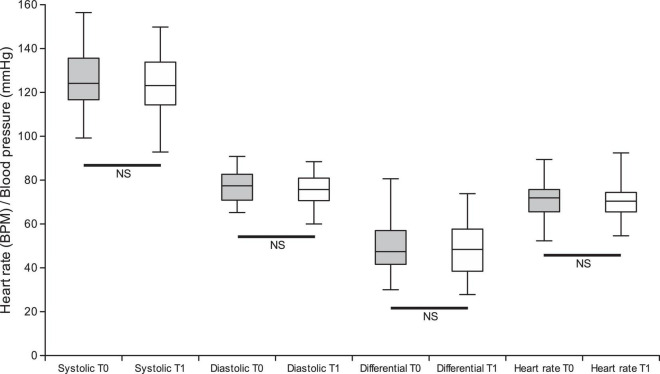
Graphical representation of comparison between cardiovascular parameters: systolic arterial pressure, diastolic arterial pressure, differential arterial pressure and heart rate measured at T0 vs. T1.

No significant changes of diastolic (*Z* = −1.2, *p* = 0.2), systolic (*Z* = −1.2, *p* = 0.2) and differential (*Z* = −0.1, *p* = 0.9) blood pressure as well as heart rate (*Z* = −0.06, *p* = 1.0) before and during nabiximols treatment (Figure 2). The number of sprays taken at T1 did not correlate with T0-T1 variation of diastolic (Rho = −0.10, *p* = 0.4), systolic (Rho = −0.13, *p* = 0.3) and differential (Rho = −0.23, *p* = 0.2) blood pressure, nor variation of heart rate (Rho = 0.07, *p* = 0.8).

## Discussion

Excluding nicotine, cannabis is the drug of abuse most commonly used by adolescents worldwide (35). Recently, however, the widespread consumption of cannabinoids has not been limited to recreational use nor to young adults. Indeed, cannabinoids are also gaining popularity among the elderly as a method of treating chronic illnesses (36).

The present study is the first to evaluate the effects of a cannabinoid drug (nabiximols) in patients with post-stroke spasticity. This drug has been widely studied in patients with multiple sclerosis (2, 3) and also in patients with amyotrophic lateral sclerosis (37), but not in stroke survivors, who are much more numerous, given the high prevalence of stroke (38). These latter patients have a worse cardiovascular risk profile, and this drug offers various advantages: it can be administered by the oromucosal route, presents a balanced THC:CBD ratio, and is probably safe from the cardiovascular point of view, since, to our knowledge, nabiximols-related stroke events have not been reported in the literature so far (39). By contrast, stroke events have been described in the case of cannabinoid intake via the inhalatory route (9). In our study, all patients underwent a preliminary cardiological evaluation and their blood pressure and heart rate were monitored daily.

Only one patient complained of tachycardia during phase 2, when on active treatment, and decided to exit the study (although tachycardia had already been recorded in phase 1, while the patient was taking placebo). As regards the differential pressure, our population behaves like the general population since we have observed that the differential blood pressure increases with age.

No cardiovascular treatment-emergent adverse drug effects emerged during nabiximols treatment, namely no significant fluctuation of blood pressure and heart rate, nor ischemic or hemorrhagic events occurred. During nabiximols treatment (T1), self-assessed blood pressure and heart rate did not change compared to the baseline condition. No patients showed significant acceleration or decrease in heart rate or change in rhythm and none required an additional ECG or cardiological evaluation during the study.

The patients reached a very variable number of sprays at T1, depending on the individual highest tolerated dose. To further investigate a possible dose-related effect of nabiximols on blood pressure and heart rate we sought a correlation between the number of sprays at T1 and cardiovascular parameter variation between T0 and T1, however, the lack of correlation confirms that, nabiximols did not affect blood pressure and heart rate in our population over 4 weeks. This may be also ascribed to the fact that oromuscosal administration allows different pharmacokinetics with fewer fluctuations in blood pressure and heart rate. Unfortunately, however, the expected improvement in spasticity in stroke patients was not found, as previously published (34). This additional result of the SativexStroke trial was that nabiximols displayed a good safety profile and was well tolerated, particularly from the cardiovascular point of view.

The main limitation of the present study is the low number of participants since it was single-center. Another limitation of the study is the fact that, as regards the cardiovascular effects of nabiximols, we considered baseline (T0) values those obtained at the beginning of the active phase, while patients had already started nabiximols, although at a very low dosage. If nabiximols effect on cardiovascular parameters was not dose-dependent and effective even while taking a small number of sprays and consequently before nabiximols could reach plasma steady state, such effect cannot be appreciated by our protocol. Further studies are needed to clarify this aspect. Blood pressure and heart rate were self-assessed by patients. This might have introduced inaccuracies related to a systematic or occasional error in parameter sampling. We tried to mitigate possible measurement errors by analyzing the average values across 5 consecutive days, although this approach, along with the requirement to measure parameters only once per day, greatly reduced the temporal resolution of outcome assessments. It is therefore obvious that short-term (minutes and hours) fluctuations of blood pressure and heart rate could not have been appreciated in this study.

## Conclusion

In conclusion, an interesting result of this pilot study is the good cardiovascular safety profile of nabiximols in patients with stroke. In these patients, the possible beneficial effect of cannabinoids, such as delaying atherosclerotic progression and inflammation, may deserve further investigation. Furthermore, because of the rapidly changing landscape of cannabis laws and marijuana use in western countries, there is a pressing need for refined policy, education of both clinicians and the public, and new research. Carefully designed, prospective, short- and long-term studies are needed to obtain conclusive data on the safety and efficacy of cannabinoid drugs.

## Data availability statement

The raw data supporting the conclusions of this article will be made available by the authors, without undue reservation.

## Ethics statement

The studies involving human participants were reviewed and approved by the Comitato Etico Regionale della Liguria. The patients/participants provided their written informed consent to participate in this study.

## Author contributions

LMa conceived and designed the study, collected data as principal investigator, performed statistical analyses, and drafted the manuscript. LP and LMo performed patients evaluation and collected data. GR performed the cardiological evaluation and revised the manuscript. AC, FF, IP, NB, and CT critically revised the work for important intellectual content. All authors approved the final version.
